# Effective Boundary Conditions and Stochastic Crack Distribution for Modelling Guided Waves Scattering by a Partially Closed Interfacial Delamination in a Laminate

**DOI:** 10.3390/ma16062415

**Published:** 2023-03-17

**Authors:** Mikhail V. Golub, Olga V. Doroshenko, Yan Gu

**Affiliations:** 1 Institute for Mathematics, Mechanics and Informatics, Kuban State University, Krasnodar 350040, Russia; 2 School of Mathematics and Statistics, Qingdao University, 308 Ning Xia Lu, Laoshan District, Qingdao 266071, China

**Keywords:** guided waves, delamination, bridged crack, effective boundary conditions, laminate, eigenfrequency, diffraction, crack distribution, interface, damage, resonance

## Abstract

Cohesive and adhesive bindings degrade during operation and maintenance even if contacting materials in a manufactured laminated structure are perfectly matched at the interfaces. Two modelling approaches for describing partially closed delaminations or imperfect contact zones, which often occurs at the interfaces, are examined and considered. To confirm the adequateness of the applicability of the effective spring boundary conditions for guided wave scattering by a finite length delamination, guided wave propagation through a damaged zone with a distribution of micro-cracks is compared with an equivalent cohesive zone model, where the spring stiffnesses for the effective boundary conditions are calculated using the properties of the considered crack distribution. Two kinds of local interfacial decohesion zones with an imperfect contact at the interfaces are considered: uniform partially closed delaminations and bridged cracks. The possibility of the employment of the effective spring boundary conditions to substitute a distribution of micro-cracks is analysed and discussed. Two algorithms of generation of a distribution of open micro-cracks providing characteristics equivalent to the effective boundary conditions are presented and examined. The influence of the characteristics of a delamination on wave characteristics (eigenfrequencies, eigenforms, transmission coefficient) is investigated for several kinds of partially closed delaminations.

## 1. Introduction

Multi-layered laminate composite materials are now widely employed in automobiles, marine vehicles, aircraft and other manufacturing industries since they have superior strength, toughness, ductility and fatigue lifetime, which improve the reliability and the durability of structures [1,2]. Even if contacting materials in a manufactured laminated structure are perfectly matched at the interfaces, the cohesive and adhesive bindings degrade during operation and maintenance, which leads to the occurrence of zones with imperfect contact. Interface damages in laminates might also arise after impacts [3,4,5]. The progress of the mismatch between the material properties of different constituents leads to the formation of micro-cracks and micro-voids at the interfaces or in the interior of the layered composite structures [6,7,8]. The interface roughness increases the strength of the joint, but since the interface is not perfectly flat, it leads to a non-uniform stress–strain state and initiates micro-cracks and delaminations. Since laminate assemblies are increasingly employed in engineering structures, studies related to bi-material interface fracture analysis [9] and the evaluation of accumulated damage or degradation in the early stages of fracture are important for industrial applications [7,10]. The characteristics of ultrasonic wave propagation are directly related to the mechanical properties of laminates [11,12,13] and therefore ultrasonic methods are among the most efficient techniques in the field of non-destructive evaluation (NDE) and structural health monitoring (SHM) for evaluating material degradation [14,15,16,17,18].

Simulation of elastic wave propagation through a fractured zone is a time-consuming and challenging procedure since it must take into account the influence of all the inhomogeneities. This problem can be solved by developing efficient improved numerical methods (e.g., [19,20,21,22,23,24,25]) or employing models, where the fractured zone with a distribution of cracks is substituted by an equivalent homogeneous or inhomogeneous effective media (e.g., [26,27]). Though some wave phenomena are not taken into account in the models incorporating homogenisation, these models are efficient and suitable for many applications [28,29,30]. If inhomogeneities or uncertainties are concentrated at the interfaces in the form of a distribution of interfacial micro-cracks or an interface roughness, the irregularities are situated randomly in the vicinity of interfaces. To simulate the propagation of seismic waves through rough boundaries between the layers with different physical properties, Khachkova et al. [31] constructed statistically equivalent models with rough interfaces and constant elastic moduli and models with random coefficients and flat interfaces, and Shi [32] derived theoretical formulae for predicting the variance of the scattering amplitude and intensity using Kirchhoff approximation for known statistical parameters of the roughness.

Baik and Thompson [33] proposed a phenomenological approach for modelling imperfect contact at interfaces with micro-cracks, which is based on the substitution of a distribution of micro-cracks by the effective spring boundary conditions (ESBCs). Henceforth, various investigators have derived analytical estimations for the stiffness of an infinite damaged interface with periodic or stochastic distributions of cracks [34,35,36,37,38,39,40] in terms of parameters of the crack distribution. The methods based on the employment of the ESBCs were validated and employed for laminates with infinite damaged interfaces [13,41] and a local partially closed delamination [42].

In this study, the applicability of the ESBCs with the relations derived for spring stiffnesses [37,38] for simulating wave propagation in laminates with a locally damaged interface is investigated. Two kinds of local interfacial decohesion zones with an imperfect contact at the interfaces are considered: partially closed delaminations [42,43,44,45] and bridged cracks [46,47,48,49]. Accurate modelling of wave scattering by an interface finite size delamination crack within the framework of the linear theory of elasticity leads to the presence of fast oscillations in the stresses and displacements near crack tips [50]. The open crack model, where faces of a crack are assumed to be stress-free, is not valid for these kinds of delaminations, so the cohesive zone model (CZM) can be applied [48,50]. The latter incorporates various models for describing delaminations, including cracks where faces are in contact near the crack tips; these are also called bridged cracks. ESBCs with non-uniform spring stiffnesses can be introduced in terms of the CZM and linear theory of elasticity to characterise a part of interface with the interruption of the stress and displacement continuity, e.g., impact-induced damage.

The aim of this study is to confirm the adequateness of the applicability of the ESBC estimations obtained in [37] for wave scattering by a finite size delamination and investigate wave scattering by partially closed delaminations. To this end, guided wave propagation through a damaged zone with a distribution of micro-cracks is compared with an equivalent CZM model, where the spring stiffnesses for the ESBCs are calculated using the properties of the considered crack distribution according to [37,51]. Carpinteri et al. [52] proposed a model where a damaged zone consists of a macro-crack and multiple micro-cracks ahead of the macro-crack tips, so that it corresponds to a bridged crack. Therefore, a similar CZM model with the ESBCs is also applied and validated for bridged cracks. Statistical modelling techniques are used to create models of distributed micro-cracks or bridge cracks. For this purpose, algorithms of the generation of a distribution of open micro-cracks providing characteristics equivalent to the ESBCs are presented and examined.

The wave scattering by delaminations described by the ESBCs has been modelled employing the boundary integral equation method (BIEM) [53,54], the semi-analytical hybrid approach (SAHA) based on the BIEM [55] and the finite element method (FEM). The main advantage of the BIEM and the SAHA is its semi-analytical nature, which allows calculation of the resonance frequencies, analysis of the wave energy trapping and localisation, simulation of separate Lamb wave mode excitation, scattering and conversion due to damage [56]. Guided wave transmission through a damaged zone is compared employing two considered approaches. The possibility of the employment of the effective boundary conditions to substitute a distribution of micro-cracks at higher frequencies is demonstrated. The effectiveness of the EBSCs for modelling crack distribution for in-plane wave motion is shown considering complex-valued eigenfrequencies and guided wave transmission in a laminate structure with a partially closed delamination of finite length.

## 2. Mathematical Models of Interface Delamination

This section consists of three parts. First, two approaches applied here to model partially delaminated zones are discussed in detail: distribution of open micro-cracks and the EBSCs. The latter includes mathematical formulation of the corresponding boundary conditions and terms used. Next, the aspects of the simulation of two classes of delamination (a uniform partially closed delamination and a bridged crack) for the two approaches are explained. Finally, two algorithms of generation of a distribution of open micro-cracks providing characteristics equivalent to the ESBCs are described. The introduced models are examined and compared in Section 4.

### 2.1. Models of Partially Closed Delaminations

**Figure 1:**
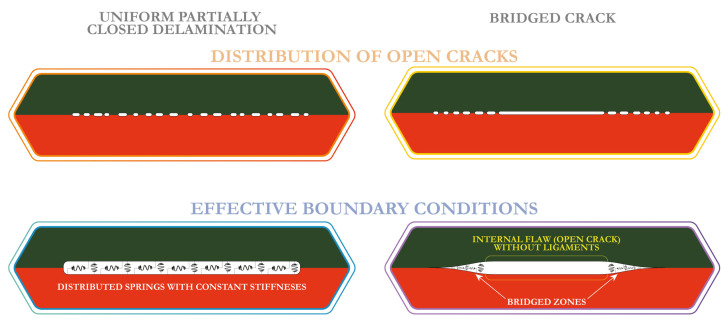
Different classes and mathematical models of delaminations considered in this study.

Following Perelmuter [57], two kinds of damaged zones or partially closed delaminations as illustrated in Figure 1 are considered in this investigation. Imperfect or weak interface, where the fracture process zone is assumed at the whole interface of two contacting materials, is the first kind of damage considered here [58,59]. The term weak interface is often employed to simulate the whole interface, whereas the prolonged zones, but of finite length, are called imperfect interfaces. Another one is the so-called bridged or cohesive crack, where the fracture process zone is assumed as a part of a delamination and the bridged zone can be comparable to the length of the flaw without ligaments and with the perfect contact outside a certain area considered as a delamination [52,60,61].

The two approaches to model two kinds of delaminations (bridged cracks and imperfect interfaces) are examined here, see Figure 1. In the first approach, a delamination is assumed to be a distribution of open micro-cracks. In the second approach, the distributed spring model with varying stiffness in a general case is employed. The latter is formulated as ESBCs

(1) $$\tau \left(x\right)=\kappa \left(x\right)\cdot \left[u\right]\;\left(x\right),\;x\in \tilde{\Omega },$$

where the traction vector $\tau =\{\sigma_{11},\sigma_{12}\}$ composed of normal and tangential stresses is assumed to be proportional to the crack opening displacement (COD) $\left[u\right]$ in a certain damaged area $\tilde{\Omega }$.

**Figure 2:**
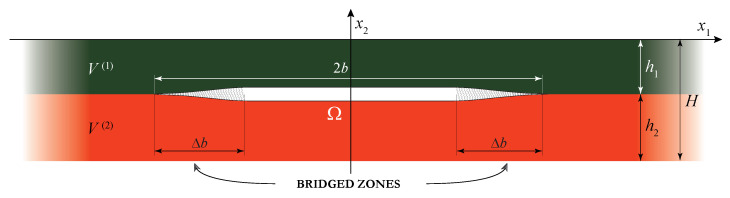
Two-layered laminate with a strip-like damaged zone including possible bridged zones.

In this study, a plain-strain problem for a laminate with a finite length part of an interface being damaged is considered. For simplicity, let us consider wave motion in a two-layered elastic waveguide $V=\cup_{j=1}^{2}V^{\left(j\right)}$ consisting of two sub-layers made of dissimilar materials of total thickness *H*. Materials of the elastic layers of thicknesses $h_{j}$ are characterised by the mass density $\rho_{j}$, Young’s moduli $E_{j}$ and Poisson’s ratios $\nu_{j}$, where j={1,2}. The damaged zone $\tilde{\Omega }=\left\{\right|x_{1}\left|\leq b,x_{2}=-h_{1}\right\}$ is situated at the interface $x_{2}=-h_{1}$ between the sub-layers. Since a uniform partially closed delamination as well as a bridged crack are considered, a general statement of the problem is formulated and the damaged zone might be split into two parts (see Figure 2): two bridged zones with varying bond stiffness $\Omega^{B}=\{b-\Delta b\leq |x_{1}\left|\leq b,x_{2}=h_{1}\right\}$ situated near the tips of the delamination and the remaining part $\tilde{\Omega }\backslash \Omega^{B}$, where a uniform bond stiffness is assumed (it also includes the case of an open crack with stress-free faces).

### 2.2. Distribution of Open Cracks

For the first kind of delamination, which is partially closed delamination, an imperfect interface is assumed in all domain $\tilde{\Omega }=\left[-b,b\right].$ According to the first approach, this damaged zone is substituted by the set of interfacial open micro-cracks $\cup_{m=1}^{M}\Omega_{m}$, both stochastically and periodically arranged. For a random distribution of micro-defects, it is assumed that the appearance of a crack at any point of the interface has a uniform distribution. The second approach involves weakening the contact between the edges of the crack in this zone and the degree of weakening is the same on $\tilde{\Omega }$, which corresponds to the constant spring stiffness (3).

The first approach for modelling a damaged zone evaluates the damage as a distribution of cracks. In this case, the damaged area $\tilde{\Omega }$ is covered by *M* intervals of lengths $2a_{m}$ corresponding to open cracks $\Omega^{\left(m\right)}\in \tilde{\Omega }$ ($\Omega^{\left(m\right)}\cap \Omega^{\left(m^{\prime }\right)}=\emptyset ,m\neq m^{\prime }$), where stress-free boundary conditions are employed

$$\sigma_{i2}\left(x\right)=0,\;x\in \Omega^{\left(m\right)},$$

while perfect contact zones are assumed in the remaining uncracked part $\tilde{\Omega }\backslash \cup_{m=1}^{M}\Omega^{\left(m\right)}$. Therefore, the crack density *C* for the damaged area $\tilde{\Omega }$ can be introduced as the ratio of length of the damaged part to lengths of the whole damaged region, i.e.,

$$C=\sum_{m=1}^{M}\frac{a_{m}}{2b}.$$

### 2.3. Effective Spring Boundary Conditions

The EBSCs in the form (1) can be used to substitute an array of open cracks (stochastic or periodically distributed). Therefore, a relation for introducing the ESBCs instead of modelling all the cracks $\Omega^{\left(m\right)}$ in the distribution is introduced in the second approach considered here. For an in-plane problem with strip-like micro-cracks, the stiffness matrix κ is diagonal, while tangential ($\kappa_{11}$) and normal ($\kappa_{22}$) stiffnesses are equal [37], i.e., $\kappa_{11}=\kappa_{22}=\kappa$. Therefore, the damage in the form of a partially closed delamination can be modelled using ESBCs with non-zero stiffnesses

(2) $$\sigma_{i2}\left(x_{1}\right)=\kappa \left(x_{1}\right)\left[u\right]\;\left(x_{1}\right),\;x_{1}\in \left[-b;b\right].$$

Stiffness value $\kappa (x_{1})$ is assumed to be spatially dependent, which allows one to describe both uniform partially closed delaminations and bridged cracks.

#### 2.3.1. Uniform Partially Closed Delamination

According to [51], the stiffness value $\kappa (x_{1})$ in (2) is a constant value if an infinite interface with an array of randomly distributed cracks of various lengths is considered. Since the appearance of a crack at any point in the interface is equiprobable, the crack density *C* is also assumed to be a constant value. By constant density, we also mean the condition that in any chosen finite domain at the interface, the ratio of the cumulative damaged zone to the domain considered is independent of the particular domain chosen. As a result, the stiffness value κ is expressed via the crack density *C*, material properties (Young’s moduli $E_{j}$ and Poisson’s ratios $\nu_{j}$) as well as the first and the second raw moments of the crack size distribution ($\bar{a}$ and $\bar{a^{2}}$ respectively):

(3) $$\kappa \equiv \frac{2}{\pi \;C}{\left(\frac{1-\nu_{j-1}^{2}}{E_{j-1}}+\frac{1-\nu_{j}^{2}}{E_{j}}\right)}^{-1}\cdot \frac{\bar{a}}{\bar{a^{2}}}=\sigma^{\kappa }\cdot \frac{\bar{a}}{\bar{a^{2}}}\cdot \frac{1}{C}.$$

#### 2.3.2. Bridged Crack

If some presuppositions for the crack distribution are used, then it makes sense to assume that the crack density *C* depends on the spatial coordinate $x_{1}$, for example, if the probability of the appearance of a larger crack in a certain sub-domain of the interface increases. In this way, the stiffness in (2) depends on the spatial variable $x_{1}$ and the relation for spring stiffness can be rewritten as follows for the class of bridged cracks considered in the present investigation [51]:

(4) $$\kappa \left(x_{1}\right)=\left\{\begin{array}{cc} \sigma^{\kappa }\cdot \frac{\bar{a}}{\bar{a^{2}}}\cdot \frac{1}{C^{B}\left(x_{1}\right)}, & b-\Delta b\leq |x_{1}|\leq b,\\ 0, & |x_{1}|<b-\Delta b, \end{array}\right.$$

Employing representation (4), the stiffness as a function of spatial coordinate $x_{1}$ is explicitly defined in terms of the crack density of micro-cracks $C(x_{1})$, which is also spatially dependent and defined in $\Omega^{B}$. To simulate delamination of the second kind related to impact-induced damages, spring stiffness is assumed to be zero in the centre and tending to infinity (corresponds to perfect contact) near the crack tips. Therefore, it is natural to assume gradual closure in the vicinity of the tips.

Here, we consider two functional relationships $C^{B}\left(x_{1}\right)$ providing the same behavior near crack tips as proposed by Goldstein and Perelmuter [61], Perelmuter [62], who proposed describing bridged cracks. If $C(x_{1})$ is a hyperbolic function introduced as follows

(5) $$C^{B}\left(x_{1}\right)=C^{\mathrm{BL}}\left(x_{1}\right)=\frac{(1-\eta )(b-\Delta b)\;b}{\Delta b\cdot |x_{1}|}+1-\frac{(1-\eta )\;b}{\Delta b},$$

the nearly hyperbolic increase in spring stiffness (4) near crack tips is achieved. To provide the force distribution in accordance with the square root law, the following relation is used for crack density:

(6) $$C^{B}\left(x_{1}\right)=C^{\mathrm{BS}}\left(x_{1}\right)=\sqrt{\frac{1-\eta^{2}}{\Delta b}}\sqrt{\frac{\eta^{2}\Delta b}{1-\eta^{2}}+b-\left|x_{1}\right|}.$$

Here, η is a small positive number regulating value of stiffness at the tips ($\eta =10^{-4}$ in the numerics presented in this study). For both functions, crack density at the edges of the bridged zone is chosen to have

$$C^{B}\left(\pm b\right)=\eta ,\;C^{B}\left(\pm b\mp \Delta b\right)=1,$$

which gives the following values for stiffness:

$$\kappa \left(\pm b\right)=\sigma^{\kappa }\cdot \frac{\bar{a}}{\bar{a^{2}}}\cdot \eta^{-1},\;\kappa \left(\pm b\pm \Delta b\right)=\sigma^{\kappa }\cdot \frac{\bar{a}}{\bar{a^{2}}}.$$

**Figure 3:**
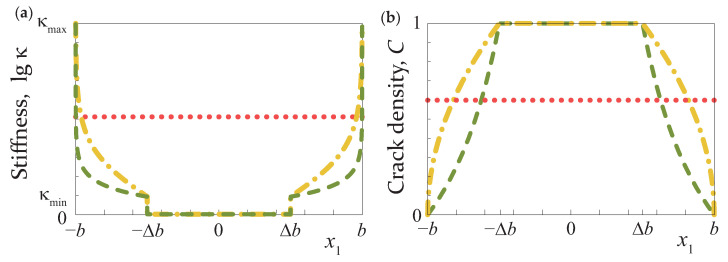
Spring stiffness distribution $\kappa (x_{1})$ (**a**) and the crack density $C(x_{1})$ (**b**) for the considered kinds of partially closed delaminations.

An example of the variation of the spring stiffness $\kappa (x_{1})$ and the crack density $C^{B}\left(x_{1}\right)$ for the two functions considered in this study (hyperbolic and square-root laws) are depicted in Figure 3. The employment of (5) and (6) allows for simulating partially closed delaminations with a bridged zone, where the crack faces interaction (fracture process zones) near the crack tips and adhesion forces are applied at the crack faces restraining the crack opening [62]. In this case, the micro-defect distribution corresponds to the presence of a central crack in $|x_{1}|\leq b-\Delta b$ and an array of cracks in $\Omega^{B}$ with decreasing length when approaching crack tips.

### 2.4. Algorithm of Open Micro-Crack Distribution Generation

#### 2.4.1. Partially Closed Delamination with Constant Crack Density (Uniform)

To simulate a partially closed delamination using crack distribution, an algorithm for the generation of the parameters of open micro-cracks $\Omega^{\left(m\right)}$ in the damaged area $\tilde{\Omega }$ is needed. For uniform partially closed delamination, a constant crack density *C* is assumed. In other words, the appearance of a micro-crack is equiprobable at any point in $\tilde{\Omega }$. At the first step, a set of *M* sample points $\chi_{m}$ uniformly distributed over the damaged zone $\tilde{\Omega }=\left[-b,b\right]$ is generated. These points are employed to initialise the appearance of open micro-cracks in a certain interval. Since the algorithm for uniform distribution assumes that the crack density *C* is constant, the lengths of micro-cracks $a_{m}$ are defined so that the cumulative length of the cracks is equal to C·2b:

(7) $$\begin{array}{c} a_{1}=\left(\frac{\chi_{1}+\chi_{2}}{2}+b\right)\cdot C;\\ a_{m}=\frac{\chi_{m+1}-\chi_{m-1}}{2}\cdot C,\;m=\bar{2,M-1}\\ a_{M}=\left(b-\frac{\chi_{M-1}+\chi_{M}}{2}\right)\cdot C. \end{array}$$

Then the coordinates for crack tips of the cracks $\Omega^{\left(m\right)}=\left\{b_{m}^{l}\leq x_{1}\leq b_{m}^{r},x_{2}=-h_{1}\right\}$ are evaluated as follows:

(8) $$\begin{array}{c} b_{1}^{l}=\chi_{1}\;\left(1-C\right)-b\cdot C;\\ b_{m}^{l}=\chi_{m}\;\left(1-\frac{1}{2}C\right)+\chi_{m+1}\cdot \frac{1}{2}C,\;m=\bar{2,M}\\ b_{m}^{r}=\chi_{m+1}\;\left(1-\frac{1}{2}C\right)+\chi_{m}\cdot \frac{1}{2}C,\;m=\bar{1,M-1}\\ b_{M}^{r}=\chi_{M}\;\left(1-C\right)+b\cdot C \end{array}$$

**Figure 4:**
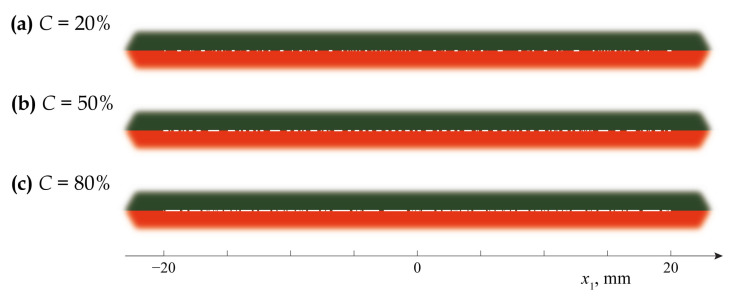
Examples of generated crack distributions for uniform partially closed delaminations with various densities: C=0.2 (**a**), C=0.5 (**b**), C=0.8 (**c**).

Figure 4 demonstrates several examples of the generation of an array of open micro-cracks via the present algorithm for three dissimilar values of the crack density *C*. For all cases, 81 micro-cracks have been generated in the damaged area $\tilde{\Omega }$.

The Kolmogorov–Smirnov test shows that the crack widths $a_{m}$ fit the beta distribution B(α,β) with parameters

$$\begin{array}{c} \alpha =\frac{\bar{a}\left(\bar{a}-{\bar{a}}^{2}-{\hat{\sigma }}_{a}^{2}\right)}{{\hat{\sigma }}_{a}^{2}},\\ \beta =\frac{\left(\bar{a}-1\right)\left(\bar{a}-{\bar{a}}^{2}+{\hat{\sigma }}_{a}^{2}\right)}{{\hat{\sigma }}_{a}^{2}}, \end{array}$$

defined in terms of the mean

$$\bar{a}=\frac{1}{M}\sum_{m=1}^{M}a_{m}$$

and the estimate of variance of a sample $\left\{a_{m}\right\}$

$${\hat{\sigma }}_{a}^{2}=\frac{1}{M-1}\sum_{m=1}^{M}{\left(a_{m}-\bar{a}\right)}^{2}.$$

**Figure 5:**
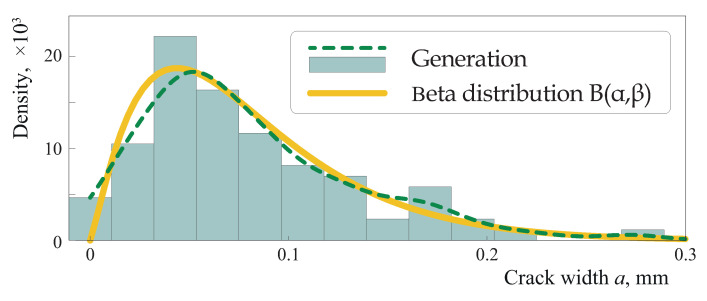
Histogram of crack width *a*.

An example illustrating how crack lengths are distributed is shown in Figure 5, where a histogram of crack widths for a single generation along with solid and dashed lines corresponding to the kernel density estimate and the theoretical density function are depicted. The kernel density estimate with a smoothing parameter h>0 is defined as follows:

$${\hat{f}}_{h}\left(x\right)=\frac{1}{Mh}\sum_{m=1}^{M}K\left(\frac{x-a_{m}}{h}\right).$$

Here, K(x) is a kernel function satisfying conditions

K(x)≥0

and

$$\int_{-\infty }^{+\infty }K\left(x\right)dx=1.$$

The routine geom_density in the R language has been employed to approximate the distribution density via the kernel smoothing method. The values of the parameter in the beta distribution for the example demonstrated in Figure 5 has been estimated as α=2.21 and β=27,564. The estimated β is rather large, and corresponds to the higher probability of the appearance of smaller cracks (less than average) being larger than the probability of the appearance of cracks larger than average.

**Figure 6:**
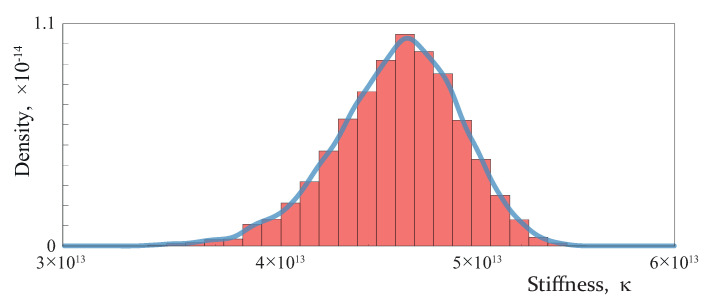
Histogram and the corresponding empirical density function of the spring stiffness κ calculated at M=81.

Further, the damaged zone [−b,b] is considered as a uniform partially closed crack with the constant spring stiffness determined according to (3). To verify the properties of the generated set of open micro-cracks, the one-way ANOVA ranks test (the Kruskal–Wallis test) was carried out for a sample of crack sizes split into four groups with respect to $x_{1}$ values. It is a non-parametric method with the null hypothesis assuming the equality of the medians of all groups. For 1000 crack generations, null hypothesis testing was performed: the null hypothesis was rejected with the confidence level 0.99 for 12% of the considered generations. Therefore, one can conclude that the algorithm consistently generates arrays of micro-cracks with a constant density in the domain $\tilde{\Omega }$ under consideration. Figure 6 shows the distribution of the spring stiffness coefficient for the crack density C=0.8, also calculated using data from 1000 generations of crack arrays. The analysis showed that the most comparable distribution among standard statistical distributions is the Weibull distribution, but the Kolmogorov–Smirnov test rejects this hypothesis with the confidence level 0.99.

#### 2.4.2. Bridged Delamination

The bridged crack considered in the present study includes a single central macro-crack dominating over micro-cracks, which are defined using an algorithm analogous to the one described in Section 2.4.1. The set of initialisation points of micro-cracks $\left\{\chi_{m},\;m=\bar{1,M}\right\}$ is generated as a sample from a uniform distribution over the interval [b−Δb,b]. The crack density function at the interval [b−Δb,b] is introduced as a piece-wise continuous function

(9) $$C\left(x_{1}\right)=\left\{\begin{array}{c} f\left(\chi_{1}\right),\;\;x_{1}\in \left[b-\Delta b,c_{1}\right),\\ f\left(\chi_{m}\right),\;x_{1}\in \left[c_{m-1},c_{m}\right),\;m=\bar{2,M-1},\\ f\left(\chi_{M}\right),\;\;x_{1}\in \left[c_{M-1},b\right], \end{array}\right.$$

where $c_{m}$ are the midpoints of the intervals $\left[\chi_{m},\chi_{m+1}\right]$. Accordingly, the crack widths are calculated via formulae similar to (7), but with the constant stiffness specified according to (9):

(10) $$\begin{array}{c} a_{1}=\left(\frac{\chi_{1}+\chi_{2}}{2}+b\right)\cdot C\left(x_{1}\right);\\ a_{m}=\frac{\chi_{m+1}-\chi_{m-1}}{2}\cdot C\left(x_{1}\right),\;m=\bar{2,M-1}\\ a_{M}=\left(b-\frac{\chi_{M-1}+\chi_{M}}{2}\right)\cdot C\left(x_{1}\right). \end{array}$$

This algorithm of the generation of a distribution of width allows to avoid crack intersections. The same algorithm is used to generate cracks in the domain [−b,−b+Δb], corresponding to the left bridged zone of the delamination.

**Figure 7:**
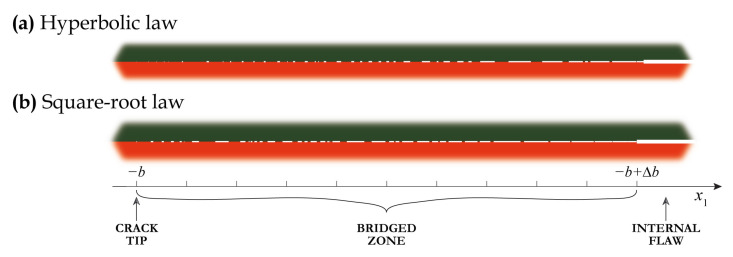
Examples of generated crack distributions for bridged cracks at b=20 mm, Δb=10 mm, $\eta =10^{-4}$: hyperbolic law (**a**) and square-root law (**b**).

**Figure 8:**
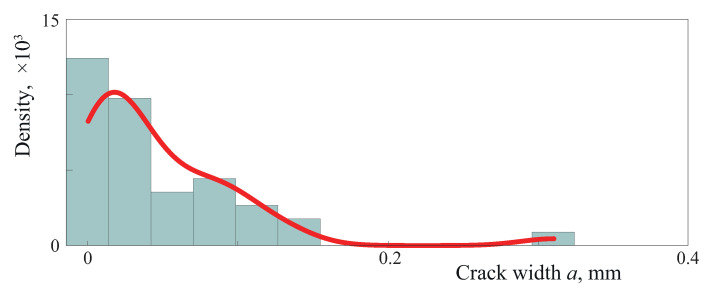
A typical example of histogram and the empirical density function of generated crack width distributions *a* in bridged zones (b=20 mm, Δb=10 mm, $\eta =10^{-4}$) for hyperbolic law.

**Figure 9:**
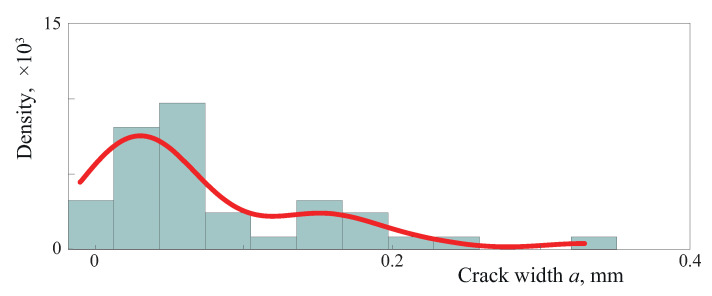
A typical example of histogram and the empirical density function of crack width distributions *a* in bridged zones (b=20 mm, Δb=10 mm, $\eta =10^{-4}$) for square-root law.

Two examples of an array of micro-cracks generated in accordance with the algorithm described above with the use of hyperbolic and square-root laws of crack density for simulating bridged cracks are depicted in Figure 7. An internal flaw occupying interval [−Δb,Δb] is shown here as a thick white line, whereas micro-cracks $\Omega^{\left(m\right)}$ situated in the left bridged zone [−b,−b+Δb] are shown as thin white intervals. One can see that the two laws lead to various proportions between larger and smaller cracks. Histograms demonstrated in Figure 8 and Figure 9 show crack width distributions *a* in bridged zone Δb=10 mm. These figures illustrate the fact that the hyperbolic law provides more smaller cracks compared to the square-root law.

## 3. Mathematical Model of a Laminate with an Interface Delamination

Let us consider steady-state motion of a two-layered waveguide of thickness *H* with an interface damage $\tilde{\Omega }$ of width *w* with the angular frequency ω=2πf described in Section 2.1. Both approaches employed to simulate damaged zones can be described by the same boundary condition (2). In the case of ESBCs, spring stiffness is calculated via (4) along with (5) or (6). Distributions of cracks can also be described by (2) if stiffness value interchanges are stress-free and boundary conditions are continuous, i.e.,

(11) $$\kappa \left(x_{1}\right)=\left\{\begin{array}{cc} 0 & x\in \Omega^{\left(m\right)},\\ \infty , & x\in \tilde{\Omega }\backslash {⋃}_{m=1}^{M}\Omega^{\left(m\right)}. \end{array}\right.$$

**Figure 10:**
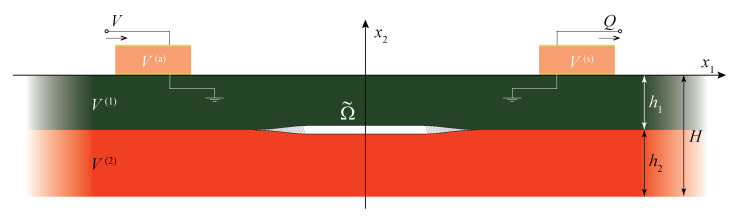
Geometry of the problem: multi-layered waveguide with a distribution of open cracks.

Two configurations are considered: damaged waveguide with and without two piezoelectric wafer active transducers (PWATs) acting as an actuator and as a sensor. Rectangular PWATs mounted at the upper surface of the waveguide occupy, respectively, domains ${\hat{V}}^{\left(a\right)}$ and ${\hat{V}}^{\left(s\right)}$. The PWATs are assumed to be of the same length *a* and thickness *d*, being situated at the same distance from the damage $\tilde{\Omega }$ with centres of PWATs located 200 mm from each other (see Figure 10). The PWATs are assumed to be made of the same piezoelectric material with the mass density $\hat{\rho }$ and the tensors of elastic, piezoelectric and dielectric constants ${\hat{C}}_{ijkl}$, ${\hat{e}}_{kij}$ and ${\hat{\varepsilon }}_{ik}$, respectively.

For time-harmonic motion with the angular frequency ω=2πf, the displacement vector $u_{j}=\left\{u_{1j},u_{2j}\right\}$ obeys the Lame equation

(12) $$\frac{1-\nu_{j}}{1-2\nu_{j}}\nabla \cdot \nabla u_{j}-\frac{1}{2}\nabla \times \left(\nabla \times u_{j}\right)+\frac{\left(1+\nu_{j}\right)\;\rho_{j}}{E_{j}}\omega^{2}u_{j}=0$$

in each sub-layer of the laminate. Hooke’s law relates the components of the stress tensor $\sigma_{ik}$ and the displacement vector u. The governing equations for the PWATs with the tensors of elastic, piezoelectric and dielectric constants ${\hat{C}}_{ijkl}$, ${\hat{e}}_{kij}$ and ${\hat{\varepsilon }}_{ik}$ and mass density $\hat{\rho }$ can be written in terms of the displacement vector $\hat{u}$ and the electric potential $\hat{\phi }$ [63]:

(13) $${\hat{C}}_{ijkl}\frac{\partial^{2}{\hat{u}}_{k}}{\partial x_{l}\partial x_{j}}+{\hat{e}}_{kij}\frac{\partial^{2}\hat{\phi }}{\partial x_{k}\partial x_{j}}+\hat{\rho }\omega^{2}{\hat{u}}_{i}=0,$$

(14) $${\hat{e}}_{ikl}\frac{\partial^{2}{\hat{u}}_{k}}{\partial x_{l}\partial x_{i}}-{\hat{\varepsilon }}_{ik}\frac{\partial^{2}\hat{\phi }}{\partial x_{k}\partial x_{i}}=0.$$

At the side boundaries ${\hat{S}}_{D}^{\left(j\right)}$ of PWATs ${\hat{V}}^{\left(j\right)}$ without electrodes, zero electric displacements are assumed

(15) $${\hat{D}}_{1}^{\left(j\right)}\left(x\right)=0,\;x\in {\hat{S}}_{D}^{\left(j\right)},\;j=\left\{a,s\right\}$$

At the lower surfaces of the PWATs ${\hat{S}}_{0}^{\left(j\right)}$, the electrode is grounded so that

(16) $$\hat{\phi }\left(x,t\right)=0,\;x\in {\hat{S}}_{0}^{\left(j\right)},\;j=\left\{a,s\right\}.$$

A given voltage $\varphi_{0}$ is applied at the actuator’s electroded upper surface ${\hat{S}}_{\phi }^{a}$:

(17) $$\hat{\phi }\left(x\right)=\varphi_{0},\;x\in {\hat{S}}_{\phi }^{a}.$$

The unknown electric potential φ at the upper electroded surface ${\hat{S}}_{\phi }^{s}$ of the sensor is constant and can be determined using the following boundary conditions:

(18) $$\begin{array}{c} \hat{\phi }\left(x\right)=\varphi ,\;x\in {\hat{S}}_{\phi }^{s},\\ \hat{Q}\left(\hat{u},\hat{\phi }\right)=\int_{{\hat{S}}_{\phi }^{s}}{\hat{D}}_{2}\left(x\right)dS=0, \end{array}$$

where $\hat{Q}\left(u,\phi \right)$ is electric charge. Moreover, the continuity of the displacement and the traction vectors in the contact areas and stress-free boundary conditions at the outer boundaries are assumed. If the eigenfrequencies are calculated, the PWATs are not included in the model and stress-free boundary conditions in the contact areas are utilised.

## 4. Numerical Analysis

The aim of this study is to compare the wave propagation characteristics estimated using two different approaches for modelling the interface damaged zone. Therefore, the results of the numerical analysis of the wave motion of a damaged multi-layered laminate, the geometry of which is depicted in Figure 10, is provided in this section. It should be mentioned that eigenfrequencies $f_{n}$ of a damaged laminate have been calculated without PWATs to have only eigenfrequencies related to the delamination itself. To solve the formulated boundary value problem for an unbounded laminate with damaged interface, the SAHA [55] and the FEM have been applied. A model, where perfectly matched layers have been employed to simulate an infinite waveguide, has been built in COMSOL Multiphysics 6.0 (COMSOL AB, Stockholm, Sweden). To verify numerical results, the calculations have also been provided the SAHA and the results have been compared with the FEM.

For the numerics below, the damaged zone of total length w=2b=40 mm in a two-layered laminate composed of two elastic layers (aluminium and steel) of $h_{1}=h_{2}=2$ mm thickness was chosen. The material properties of elastic and piezoelectric materials are given in Table 1 and Table 2. The PWATs are of 10 mm length and 0.2 mm thickness.

Since two approaches to modelling a partially closed delamination are employed, some parameters for boundary conditions (3) or (4) must be estimated prior to providing simulations employing the EBSCs. Thus, for a uniform partially closed delamination, the crack density *C* was first chosen to generate a crack distribution. Then, values $\bar{a}$ and $\bar{a^{2}}$ were calculated for the generated crack distribution with a given crack density *C*. The value denoted $\sigma^{\kappa }$ depends on material properties only and $\sigma^{\kappa }=36.25$ TPa for the aluminium/steel pair. Thus, spring stiffness κ equals 2.174, 0.335 and 0.128 PPa, respectively, for the crack density C= 0.2, 0.5 and 0.8. A similar procedure performed for bridged cracks gives κ(±b∓Δb)=5 PPa/m and κ(±b∓Δb)=5.987 PPa/m for the hyperbolic and the square-root laws, respectively.

### 4.1. Uniformly Partially Closed Delamination

At first, let us consider wave scattering by a uniform partially closed delamination modelled using the ESBCs with a constant crack density in the damaged part of the interface $\tilde{\Omega }$. The uniform distribution of cracks or periodic array of *M* open micro-cracks of length Cw/M mm, which also corresponds to the uniform distribution, is situated at the interface and the stiffness is calculated according to (3).

**Figure 11:**
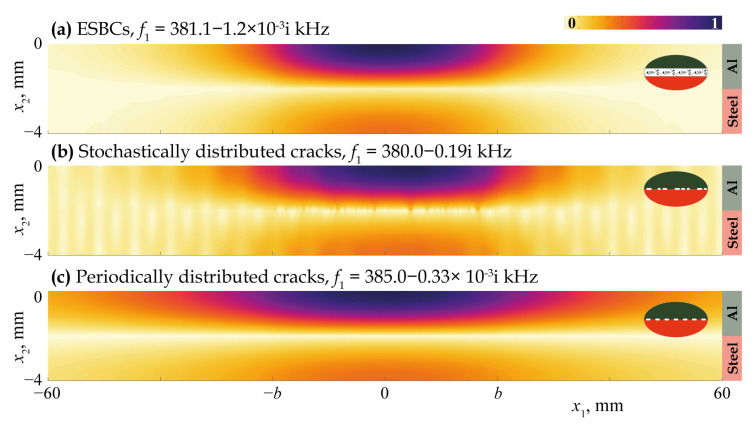
Eigenforms $\left|u|(x_{1},x_{2}\right)$ related to eigenfrequencies $f_{1}$ for a uniform partially closed delamination of length w=40 mm with the crack density C=0.5 modelled as an array of stochastically (**b**) and periodically (**c**) distributed cracks and via the ESBCs (**a**).

**Figure 12:**
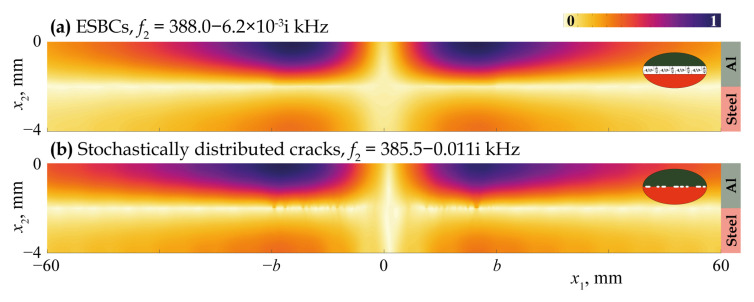
Eigenforms $\left|u|(x_{1},x_{2}\right)$ related to eigenfrequencies $f_{2}$ for a uniform partially closed delamination of length w=40 mm with the crack density C=0.5 modelled as an array stochastically (**b**) and via the ESBCs (**a**).

**Figure 13:**
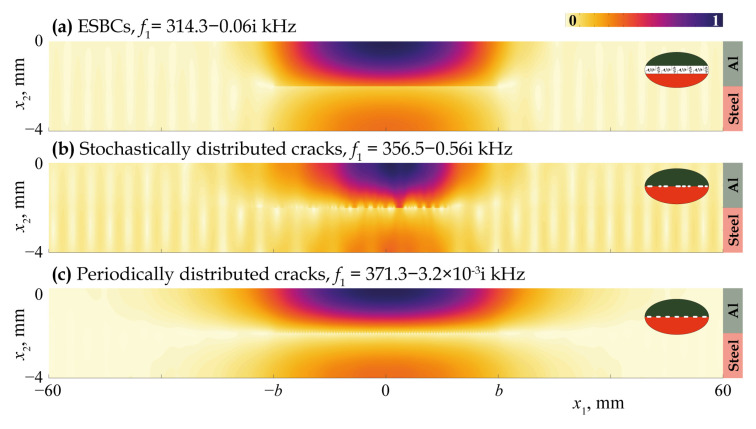
Eigenforms $\left|u|(x_{1},x_{2}\right)$ related to eigenfrequencies $f_{1}$ for a uniform partially closed delamination of length w=40 mm with the crack density C=0.8 modelled as an array of stochastically (**b**) and periodically (**c**) distributed cracks and via the ESBCs (**a**).

**Figure 14:**
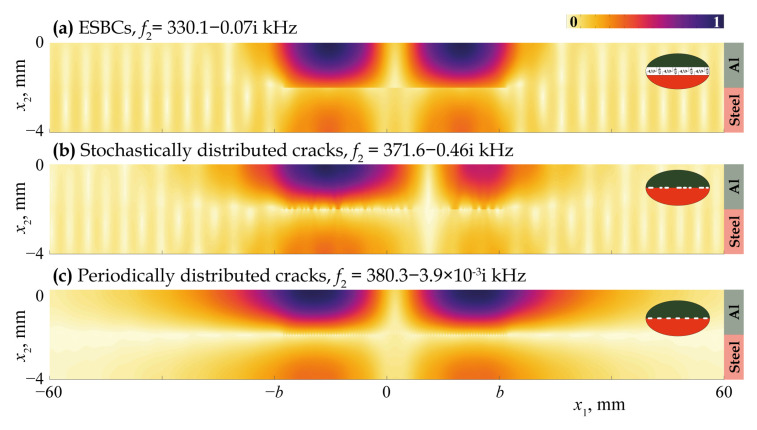
Eigenforms $\left|u|(x_{1},x_{2}\right)$ related to eigenfrequencies $f_{2}$ for a uniform partially closed delamination of length w=40 mm with the crack density C=0.8 modelled as an array of stochastically (**b**) and periodically (**c**) distributed cracks and via the ESBCs (**a**).

**Figure 15:**
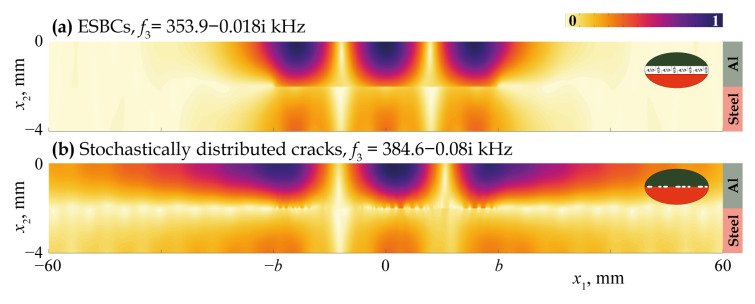
Eigenforms $\left|u|(x_{1},x_{2}\right)$ related to eigenfrequencies $f_{3}$ for a uniform partially closed delamination of length w=40 mm with the crack density C=0.8 modelled as an array stochastically (**b**) and via the ESBCs (**a**).

Figure 11, Figure 12, Figure 13, Figure 14 and Figure 15 demonstrate the eigenfrequencies and the corresponding eigenforms $\left|u|(x_{1},x_{2}\right)$ calculated for the first eigenfrequencies $f_{n}$ situated near the real axis (Imf=0) in the complex frequency plane *f* for a uniform partially closed delamination of length w=40 mm if the crack density C=0.5 and C=0.8. Here, eigenforms for random and periodic crack distributions with the same crack density *C* are compared with eigenforms for damage modelled via the ESBCs. The eigenfrequencies and eigenforms calculated for a random distribution and for the ESBCs are in better agreement with each other compared to a periodic array. It should also be noted that the second eigenfrequency for C=0.5 and the third eigenfrequency for C=0.8 corresponding to the trapping and wave localisation in the vicinity of the damaged zone were not found numerically for periodic array in the frequency range f∈[0,400] kHz.

**Figure 16:**
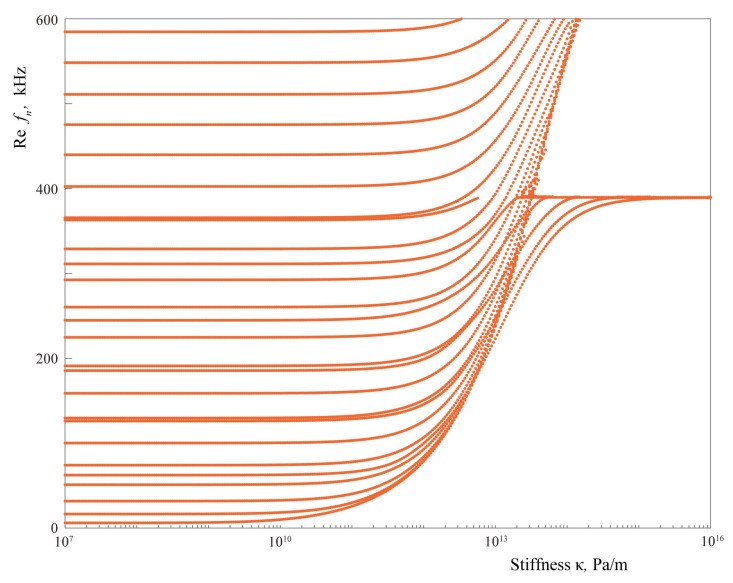
Eigenfrequencies $f_{n}\left(\kappa \right)$ with imaginary part |Imfn|≤2 kHz of the laminate with an interfacial uniform partially closed delamination of length w=40 mm modelled using ESBCs.

The analysis of eigenfrequency values given in Figure 11, Figure 12, Figure 13, Figure 14 and Figure 15 shows that the real parts of eigenfrequencies increase with the increase in the spring stiffness value κ. To investigate the influence of spring stiffness, eigenfrequency dependency $f_{n}\left(\kappa \right)$ was calculated, which is demonstrated in Figure 16. One can see that stiffness value affects sufficiently eigenfrequencies only for $\kappa >10^{10}$ Pa/m, whereas the eigenfrequencies and the corresponding eigenforms for $\kappa <10^{9}$ Pa/m almost fully coincide with the case of open crack of the same length. The imaginary parts of most eigenfrequencies increase with κ growth, which is also in agreement with the results of Eriksson [64] for partially closed circular delaminations in an elastic space. For stiffness values $\kappa >10^{13}$ Pa/m, which might be associated with a crack distribution with realistic parameters in accordance with the assumptions made for introducing EBSCs [37], real parts of all eigenfrequencies become greater than 200 kHz.

**Figure 17:**
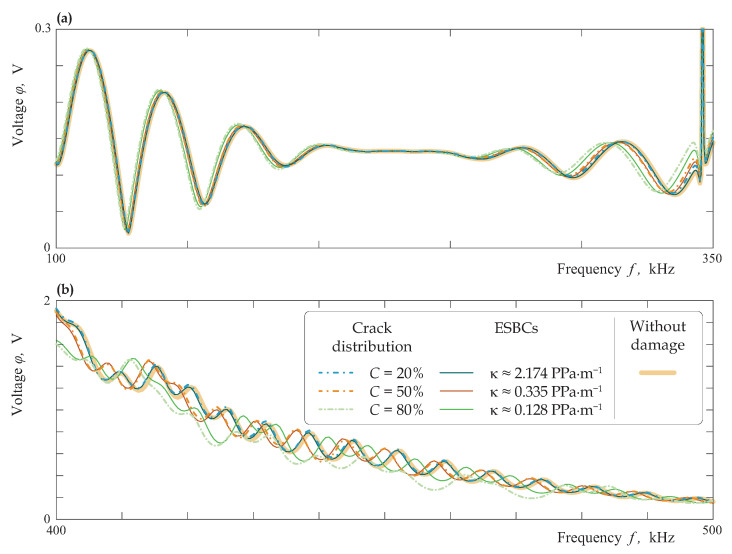
Voltage |φ|(f) sensed by PWAT at the surface of the two-layered laminate with interfacial damaged zone of length w=40 mm modelled as an array stochastically (dash-dotted lines) and via the ESBCs (thin solid lines) at lower (**a**) and higher (**b**) frequencies.

The stiffness influence is investigated considering Lamb wave transmission sensed by the PWAT behind the damaged zone, i.e., voltage φ(f) sensed by PWAT at the surface is analysed. Figure 17 depicts the frequency dependencies of $\varkappa^{+}\left(f\right)$ for a laminate with interfacial damaged zone of length w=40 mm modelled as an array stochastically (dash-dotted lines) and via the ESBCs (thin solid lines) at three different values of crack density *C*. Moreover, voltage measured in a laminate without damage is exhibited as a thick solid line. At low frequencies below 100 kHz, the measured voltage is almost the same for all kinds of damage. In the frequency range f∈[100,300] kHz, only the scattering by the severest damage at C=0.8 causes a significant difference with the undamaged state, while the observed difference in φ value for C=0.5 and C=0.2 is not so essential. The measured voltage differs significantly for C=0.8 and C=0.5 at higher frequencies larger than the third cut-off frequency (f≥400 kHz). Two approaches provide very similar results except C=0.8 at f≥400 kHz, which can be explained by the assumptions made during the ESBC derivations. Indeed, the ESBC model has some limitations and it is weakly applicable for the severest damage when neighbouring cracks interact with each other and higher frequencies, i.e., for large values of the crack density *C* and the frequency *f*.

### 4.2. Bridged Crack

**Figure 18:**
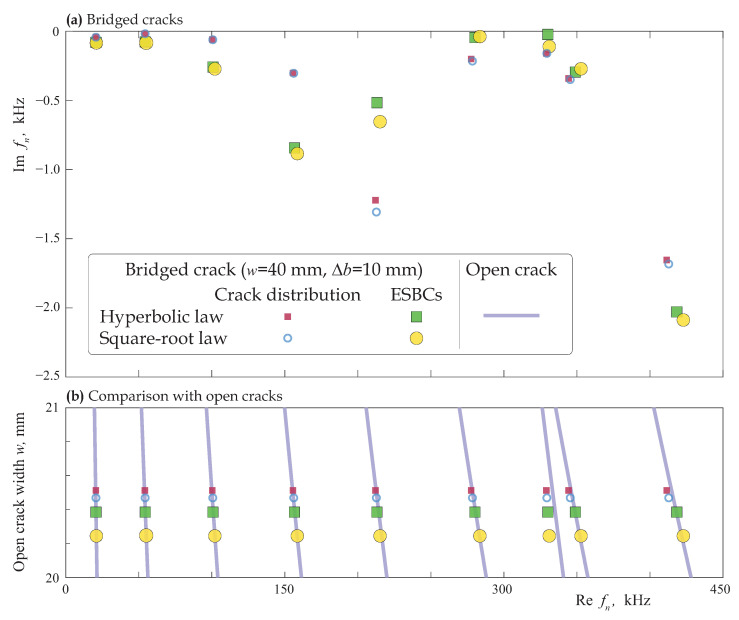
Eigenfrequencies $f_{n}\left(w\right)$ of the laminate with an open crack (**a**) and eigenfrequencies $f_{n}$ of the laminate with an interfacial bridged crack of length w=40 mm with two bridged zones of Δb=10 mm modelled as an array of cracks and via the ESBCs using the hyperbolic and square-root laws (**b**).

To analyse the influence of adhesion or bridged zones near the crack tips for bridged cracks, eigenfrequencies of delamination modelled via relations (4) and (11) were computed, respectively, for crack distribution and employing ESBCs. Figure 18a exhibits the eigenfrequencies for a bridged crack of length w=40 mm with bridged zones of Δb= 10 mm width modelled employing two approaches and two laws (hyperbolic and square-root). One can see that the four eigenfrequencies are situated quite close to each other (the relative difference is smaller than 1–2%); the eigenforms, which are not provided in the paper, are also almost the same for the four considered models of delaminated zones. It should also be noted that the eigenfrequencies estimated using the same approach are situated closer to each other.

Figure 18b demonstrates the dependence of the real part of eigenfrequencies $f_{n}\left(w\right)$ of an open crack on its length. To compare with the case of bridged crack, the eigenfrequencies calculated for the bridged crack as described in the previous paragraph are also shown in Figure 18b, where markers show the open crack length with the best fit to eigenfrequencies of the corresponding bridged crack. A good agreement between a bridged crack and an open crack of length smaller than 2b is observed for all the four considered cases, but the estimated open crack length corresponding to close values of $f_{n}$ is different, the estimated length varies from 20.2 mm to 20.5 mm. It should be noted that eigenfrequencies of a bridged crack do not perfectly fit the eigenfrequencies of any open crack.

**Figure 19:**
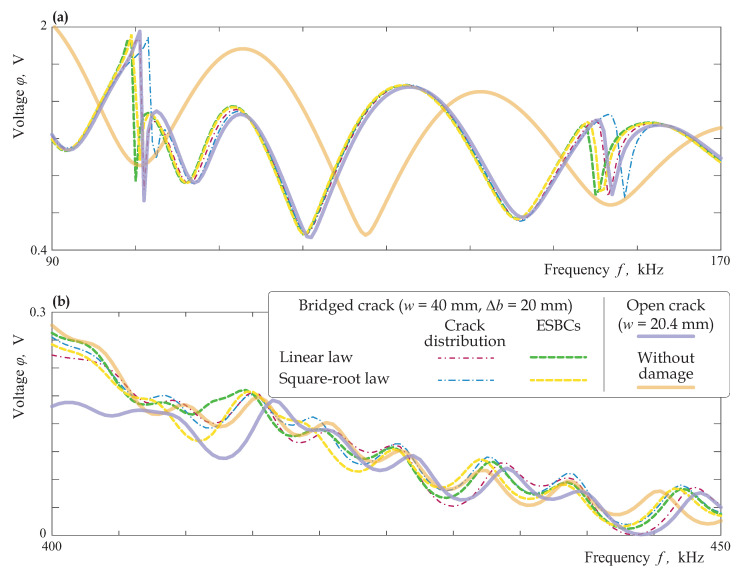
Voltage φ(f) sensed by PWAT at the surface of the laminate with interfacial bridged crack of length w=40 mm (Δb=20 mm) modelled as an array of open micro-cracks and via the ESBCs at lower (**a**) and higher (**b**) frequencies.

Sensor voltage φ(f) measured at the second PWAT was analysed for the same four bridged cracks and for an open crack of w=20.4 mm width in the same manner as for uniform partially closed delaminations, cf. Figure 17. The voltage at the sensor for the considered case of bridged cracks is sufficiently different from the voltage for undamaged laminate, see Figure 19. The results for the four models of bridged cracks and for an open crack are quite similar for lower frequencies below 120 kHz except the frequency ranges near eigenfrequencies (resonance peaks are somehow shifted from each other). At the higher frequencies larger than the third cut-off frequency (f≥400 kHz), the discrepancy between the five considered models increases, but the plots φ(f) are still qualitatively similar.

## 5. Discussion

The cohesive and adhesive bindings degrade during operation and maintenance, even if contacting materials in a manufactured laminated structure are perfectly matched at the interfaces. Therefore, the analysis of the dynamic behavior of partially damaged interfaces between elastic materials is of significant importance, especially for damage identification in composite materials and bonded joints using ultrasonic waves [65]. The configuration and optimisation of guided wave-based NDT/SHM systems for damage detection requires sufficient computational resources, especially when partially closed delaminations are to be taken into account and identified.

In this paper, it is demonstrated that the effective spring boundary conditions proposed by Baik and Thompson [33] and specified in [37,38] for an infinite number of micro-cracks at the whole unbounded interface can be efficiently applied for simulation of various distributions of interfacial micro-cracks situated in a finite part of an interface. The possibility of the employment of the EBSCs has been demonstrated for two kinds of local interfacial decohesion zones: uniform partially closed delaminations and bridged cracks.

The authors suppose further extension of the techniques and approaches developed in this investigation. To reduce the computational costs, an effective model is proposed and validated here for in-plane problems, but the approaches can be applied and developed for the three-dimensional problems employing relations for spring stiffnesses derived by Lekesiz et al. [39], Golub and Doroshenko [40], Lekesiz et al. [66], Golub et al. [67]. Thus, Zhang et al. [68] proposed an ultrasonic method providing an accurate crack evaluation and reconstructing a shape of an open crack and the employment of such procedures along with the EBCSs might be further extended to be also efficient for the identification of bridged cracks and zones with imperfect contact.

In addition, the EBSCs and can be employed for modelling partially closed delaminations in carbon fibre reinforced polymer composites made of anisotropic/orthotropic layers, which have increasingly become the material of choice for design of the main load carrying components in advanced structures over recent decades. An experimental validation of the theoretical results presented in this study could be provided using the fabrication techniques employed in [13,69] to manufacture laminates with damaged interfaces.

## Figures and Tables

**Table 1:** The material properties.

Material	Poisson’s Ratio, ν	Young’s Modulus, *E* (GPa)	Density ρ (kg/m^3^)
Aluminium	0.33	70	2700
Steel	0.17	74	7900

**Table 2:** Material properties of PWATs.

Material	Elastic Constants (GPa)	Piezoelectric Constants (C/m^2^)	Dielectric Constants $10^{-9}$(F/m)	Density (kg/m^3^)
Piezoeletric	${\hat{C}}_{1111}=120$	${\hat{e}}_{211}=-7.24$	${\hat{\varepsilon }}_{11}=9.12$	7800
material	${\hat{C}}_{1112}=67.1$	${\hat{e}}_{212}=13.77$	${\hat{\varepsilon }}_{22}=7.55$	
PWTs	${\hat{C}}_{2222}=94.2$	${\hat{e}}_{112}=11.91$		
(PIC 155)	${\hat{C}}_{1212}=22.3$			

## Data Availability

Not applicable.
